# TMT Quantitative Proteomics Reveals the Molecular Mechanism Behind Meat Quality Changes in Nile Tilapia Exposed to Environmental Concentrations of Microcystin-LR

**DOI:** 10.3390/toxins18010039

**Published:** 2026-01-12

**Authors:** Yichao Li, Huarong Xiao, Jun Xie, Liping Liu, Fajun Jiang, Jingqiu Liao, Ermeng Yu

**Affiliations:** 1 Guangxi Key Laboratory of Marine Environmental Science, Guangxi Academy of Marine Sciences, Guangxi Academy of Sciences, Nanning 530007, China; 2 China-ASEAN Belt and Road Joint Laboratory on Mariculture Technology (Shanghai), Shanghai Ocean University, Shanghai 201306, China; 3 Pearl River Fisheries Research Institute, Chinese Academy of Fishery Sciences, Guangzhou 510380, China

**Keywords:** MC-LR, meat quality, proteomics, oxidative stress, protein synthesis

## Abstract

This study investigated the effects of chronic MC-LR exposure (0 μg/L [Control], 1 μg/L [M1], 3 μg/L [M3], 10 μg/L [M10], and 30 μg/L [M30]) on the muscle nutrient composition, meat quality, and muscle proteomic profile of Nile tilapia (*Oreochromis niloticus*). In the high-dose group (M30), MC-LR exposure compromised the muscle antioxidant status of Nile tilapia, resulting in reduced meat quality, as evidenced by decreased pH value and water-holding capacity, elevated lipid/protein oxidation, and altered texture parameters (shear force and fragmentation index). Proteomic analysis further revealed a downregulation of proteins associated with ribosomes, suggesting an impairment of muscle protein synthesis in the M30 group. Moreover, despite chronic exposure, only low levels of MC-LR accumulated in the muscle tissue, indicating a negligible health risk to consumers. Collectively, these findings offered valuable insights into the impact of environmental MC-LR contamination on fish muscle quality and nutritional value.

## 1. Introduction

Nowadays, fish meat has gained popularity in global diets due to its high nutritional value, which includes rich sources of protein, essential amino acids, and polyunsaturated fatty acids (PUFAs). The consumption of aquatic food increased from 9.9 kg in the 1960s to 20.7 kg in 2022, with aquatic products now supplying over 20% of global animal protein intake [1]. As the predominant component of world fisheries production (exceeding 50%), fish represent the most significant source of aquatic animal protein [1]. Beyond genetic and feed factors, fish muscle quality—encompassing nutritional composition, texture, and flavor—is significantly influenced by external environmental conditions such as dissolved oxygen, temperature, and nitrogenous compounds [2,3,4]. For instance, ammonia exposure has been reported to reduce umami, water-holding capacity, and the content of sweet amino acids and PUFAs in rainbow trout, thereby compromising its muscle quality [5]. Similarly, acute nitrite stress was found to increase the pH and alter the texture of Wuchang bream [6], while chronic exposure to erythromycin disrupted muscle metabolism and diminished flavor attributes (e.g., umami and sweetness) in turbot (*Scophthalmus maximus*) [7]. These findings collectively underscore that environmental factors are critical determinants of fish muscle quality.

The proliferation of cyanobacterial blooms, driven by water eutrophication and climate change, has raised significant public health concerns due to the associated release of cyanotoxins, particularly microcystins (MCs) [8]. Among them, MC-LR is recognized as the most prevalent and perilous variant of the microcystins, owing to its high toxicity [9]. Previous studies have indicated that the concentrations of dissolved MCs in natural water and aquaculture ponds typically range from 0.1 to 10 µg/L, but these levels could escalate to 30 µg/L during algal bloom seasons [10,11]. However, during severe cyanobacterial bloom events (e.g., summer and autumn in temperate regions, or rainy seasons in subtropical areas), MC-LR concentrations can rapidly escalate to 30 µg/L in eutrophic lakes, reservoirs, and intensive aquaculture ponds [12]. For instance, Wang et al. (2010) reported that MC-LR concentrations reached 35.8 µg/L in Lake Taihu (China) during a massive Microcystis bloom, with such high levels persisting for 2–4 weeks [13]. Microcystin concentrations were 2.19–39.60 μg/L and 15.22–128.14 μg/L in the ponds at Sankuldhara and Lakshmikund, respectively [14]. These observations confirm that 30 µg/L is not only environmentally relevant but also represents a conservative upper limit of realistic exposure in eutrophic aquaculture systems—an increasingly common scenario due to global warming and excessive nutrient input (e.g., from agricultural runoff or high-density feeding) [15]. Notably, bloom dynamics exhibit significant temporal and spatial variability: MC-LR concentrations peak during the stationary phase of cyanobacterial growth (usually 2–3 weeks after bloom initiation) and decline gradually as blooms collapse, but recurrent low-to-moderate blooms can lead to prolonged exposure (1–3 months) to MC-LR concentrations > 10 µg/L in aquaculture settings [12,14]. MCs enter fish organisms through the food chain or direct exposure, accumulating in various tissues with prolonged retention owing to their metabolic stability, which constitutes a potential human health hazard [16].

Moreover, numerous studies have previously explored the impact of MC-LR on liver toxicity, enterotoxicity, and neurotoxicity in fish [9]. A foundational review by Malbrouck and Kestemont (2006) comprehensively summarized the key toxic effects of microcystins in fish, including oxidative stress, growth impairment, and tissue-level damage [17]. However, the muscle toxicity of MC-LR has received relatively limited attention. Notably, acute MC-LR exposure (1 μg/L) for 96 h induced morphological changes and inflammation of muscle in *Astyanax altiparanae* [18]. Acute 96 h exposure to MC-LR also triggered significant alterations in myopathy-related proteins in the muscle tissues of zebrafish [19]. Furthermore, limited information is known about the impact of chronic exposure to low concentrations of MC-LR on muscle quality, and the underlying toxic mechanisms in fish, particularly at environmentally relevant concentrations, are poorly understood. Therefore, it is imperative to investigate the ecotoxicological effects of chronic MC-LR exposure at environmental concentrations on fish muscle and clarify the underlying molecular mechanism. Nile tilapia (*Oreochromis niloticus*) is a major commercial fish with a global production of approximately 4.5 million tons, ranking third in global finfish production [1]. As one of the most extensively farmed fish, Nile tilapia faces a high risk of MC exposure. Therefore, this study evaluated the effects of chronic exposure to environmentally relevant MC-LR concentrations on the quality and safety of Nile tilapia muscle, and to further explore the molecular mechanisms driving muscle quality changes via proteomic analysis. These findings are expected to contribute to improving the safety of Nile tilapia for human consumption and optimizing its aquaculture practices.

In this study, the residual risks in Nile tilapia muscle and changes in muscle quality and biochemical parameters were first assessed. Subsequently, we investigated differentially expressed proteins and pathways using TMT (Tandem Mass Tag)-based quantitative proteomics to elucidate the potential mechanisms underlying MC-LR-induced stress. Our results offered critical insights into the meat quality changes and molecular mechanisms associated with chronic MC-LR exposure, thereby acting as a crucial reference for assessing the muscle quality and safety risk assessments of fish exposed to environmental toxins.

## 2. Results

### 2.1. The Flesh Quality Parameters

**Figure 1:**
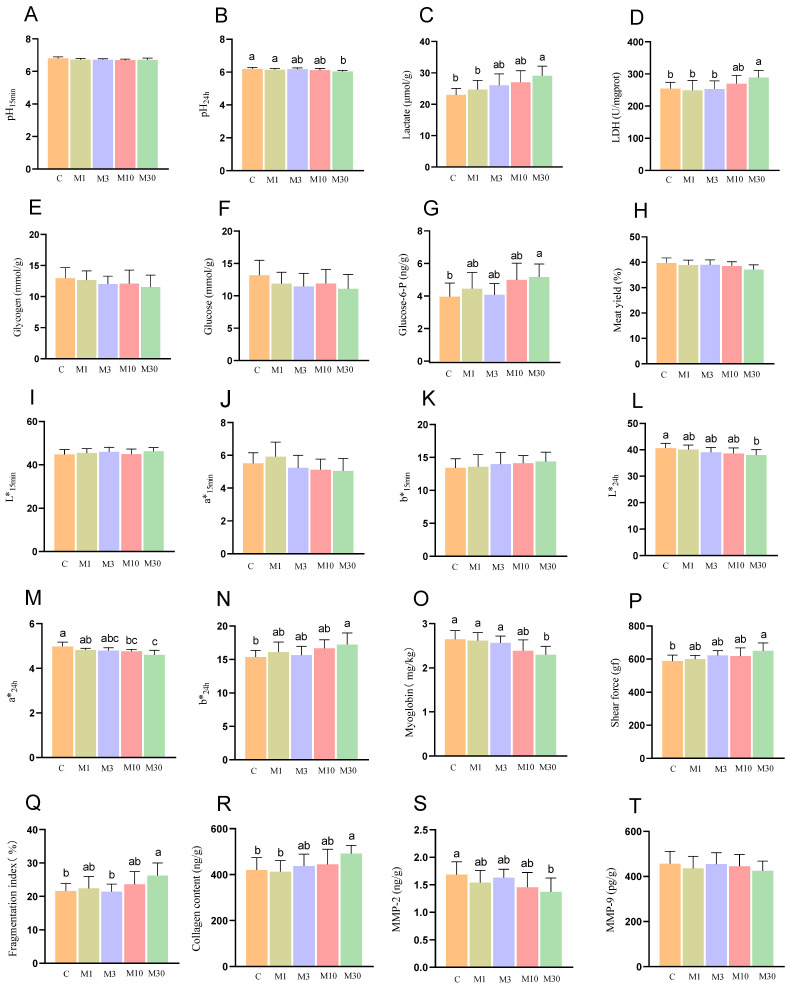
Effects of chronic MC-LR exposure on the meat quality parameters of Nile tilapia. (**A**), pH 15 min; (**B**) pH 24 h; (**C**), lactate; (**D**) lactate dehydrogenase; (**E**) glycogen; (**F**) glucose; (**G**) glucose-6-P; (**H**) meat yield; (**I**) L* 15 min; (**J**) a* 15 min; (**K**) b* 15 min; (**L**) L* 24 h; (**M**) a* 24 h; (**N**) b* 24 h; (**O**) myoglobin; (**P**) shear force; (**Q**) fragmentation index; (**R**) collagen content; (**S**) matrix metalloproteinase 2; (**T**) matrix metalloproteinase 9. Values of same parameters with different letters were significantly different (*p* < 0.05, *n* = 6). Meat yield (%) = [(Body weight − Head weight − Fin weight − Visceral weight − Bone weight)/Body weight] × 100. Data was presented as means ± SD. C, control, 0 μg/L MC-LR; M1, 1 μg/L MC-LR; M3, 3 μg/L MC-LR; M10, 10 μg/L MC-LR; M30, 30 μg/L MC-LR.

As shown in Figure 1, compared with the control, the M30 group exhibited a lower pH value at 24 h, along with higher LDH activity and increased levels of lactate and glucose-6-P (*p* < 0.05) (Figure 1B–D,G), indicating enhanced glycolytic potential during the post-slaughter period. Meanwhile, chronic MC-LR exposure affected the meat color properties of Nile tilapia, as illustrated by a decreased a* value and L* value at 24 h in the M10 and M30 groups as well as an increased b* value at 24 h in the M30 group (*p* < 0.05) (Figure 1L–N). Moreover, the M30 group displayed lower myoglobin content and MMP-2 activity relative to the control (*p* < 0.05) (Figure 1O,S). In addition, the M30 group demonstrated a higher shear force, fragmentation index, and collagen content compared to the control (*p* < 0.05) (Figure 1P–R).

### 2.2. Water-Holding Capability

**Figure 2:**
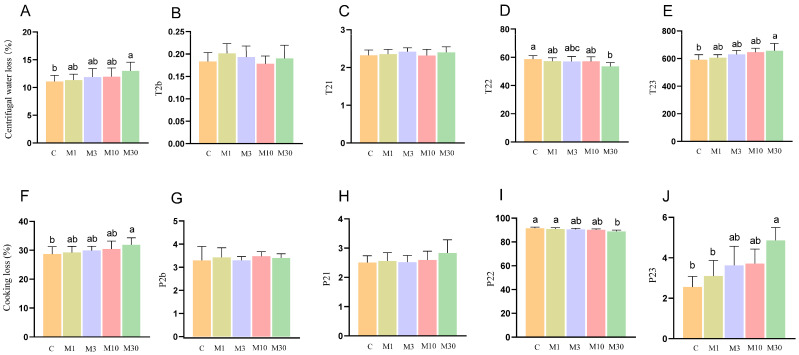
Effects of chronic MC-LR exposure on the water-holding capability of Nile tilapia muscle. (**A**) Centrifugal water loss; (**B**–**E**) distribution of T2 relaxation times; (**F**) cooking loss; (**G**–**J**) corresponding T2 peak area ratio-P2. Data was presented as means ± SD. Values of same parameters with different letters were significantly different (*p* < 0.05, *n* = 6). C, control, 0 μg/L. MC-LR; M1, 1 μg/L MC-LR; M3, 3 μg/L MC-LR; M10, 10 μg/L MC-LR; M30, 30 μg/L MC-LR.

As shown in Figure 2A,F, high-dose (M30) MC-LR exposure led to increased centrifugal water loss and cooking loss compared with the control (*p* < 0.05). In addition, the results of LF-NMR showed that decreased relaxation time T22 (>100 ms) and peak area ratio of T22 were observed in the M30 group (*p* < 0.05) (Figure 2D,I), whereas relaxation time T23 and the corresponding peak area ratio showed opposite trends (*p* < 0.05) (Figure 2E,J), indicating the increased proportion of free water and decreased proportion of immobilized water.

### 2.3. Antioxidant Status

**Figure 3:**
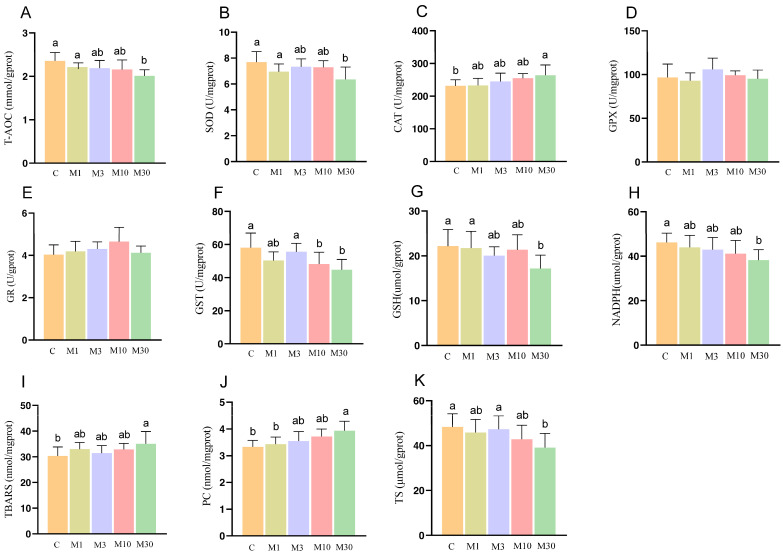
Effects of chronic MC-LR exposure on the antioxidant status of Nile tilapia muscle. (**A**) T-AOC, total antioxidant capability; (**B**) SOD, superoxide dismutase; (**C**) CAT, catalase; (**D**) GPX, glutathione peroxidase; (**E**) GR, glutathione reductase; (**F**) GST, glutathione S-transferase; (**G**) GSH, glutathione; (**H**) NADPH, nicotinamide adenine dinucleotide phosphate; (**I**) TBARS, thiobarbituric acid reactive species; (**J**) PC, protein carbonyl; (**K**) TS, total sulfhydryl. Data was presented as means ± SD. Values of same parameters with different letters were significantly different (*p* < 0.05, *n* = 6). C, control, 0 μg/L MC-LR; M1, 1 μg/L MC-LR; M3, 3 μg/L MC-LR; M10, 10 μg/L MC-LR; M30, 30 μg/L MC-LR.

Compared with the control, the M30 group exhibited lower activities of T-AOC and SOD, along with lower levels of GSH, NADPH, and TS (*p* < 0.05) (Figure 3A,B,G,H,K). In addition, MC-LR exposure lowered GST activity in the M10 and M30 groups (*p* < 0.05) (Figure 3F). These results suggested that GST is more sensitive, while broader antioxidant collapse occurs at M30. Moreover, the M30 group exhibited increased CAT activity and an increase in the values of TBARS and PC (*p* < 0.05) (Figure 3C,I,J).

### 2.4. Myofiber Histological Observation

**Figure 4:**
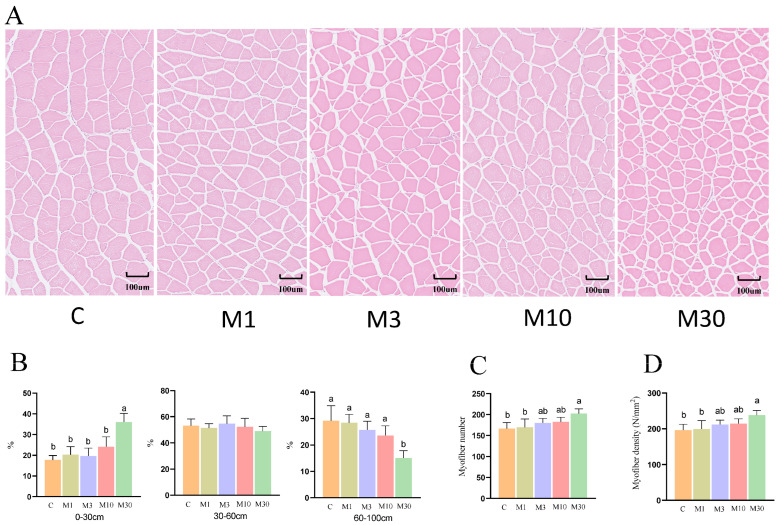
Effects of chronic MC-LR exposure on the histological characteristics of Nile tilapia muscle. (**A**) Microstructure of Nile tilapia muscle; (**B**) myofiber diameter distribution; (**C**) myofiber number under a field of view of 0.85 mm^2^ area; (**D**) myofiber density under a field of view of 0.85 mm^2^ area. Data was presented as means ± SD. Values of same parameters with different letters were significantly different (*p* < 0.05, *n* = 6). C, control, 0 μg/L MC-LR; M1, 1 μg/L MC-LR; M3, 3 μg/L MC-LR; M10, 10 μg/L MC-LR; M30, 30 μg/L MC-LR.

Compared to the control, MC-LR exposure lowered the myofiber diameter in the M30 group, as demonstrated by the increased proportion of smaller myofiber (0–30 μm) and the tdecreased proportion of larger myofiber in the M30 group (*p* < 0.05) (Figure 4A,B), suggesting inhibited muscle growth induced by MC-LR exposure. Furthermore, the M30 group showed a higher myofiber number and increased myofiber density relative to the control (*p* < 0.05) (Figure 4C,D).

Given that the M30 group demonstrated the most significant alterations across the majority of measured endpoints, muscle samples from both the M30 and control groups were subjected to proteomic analysis to further elucidate the molecular mechanisms underlying the observed changes in meat.

### 2.5. Proteomic Analysis

**Figure 5:**
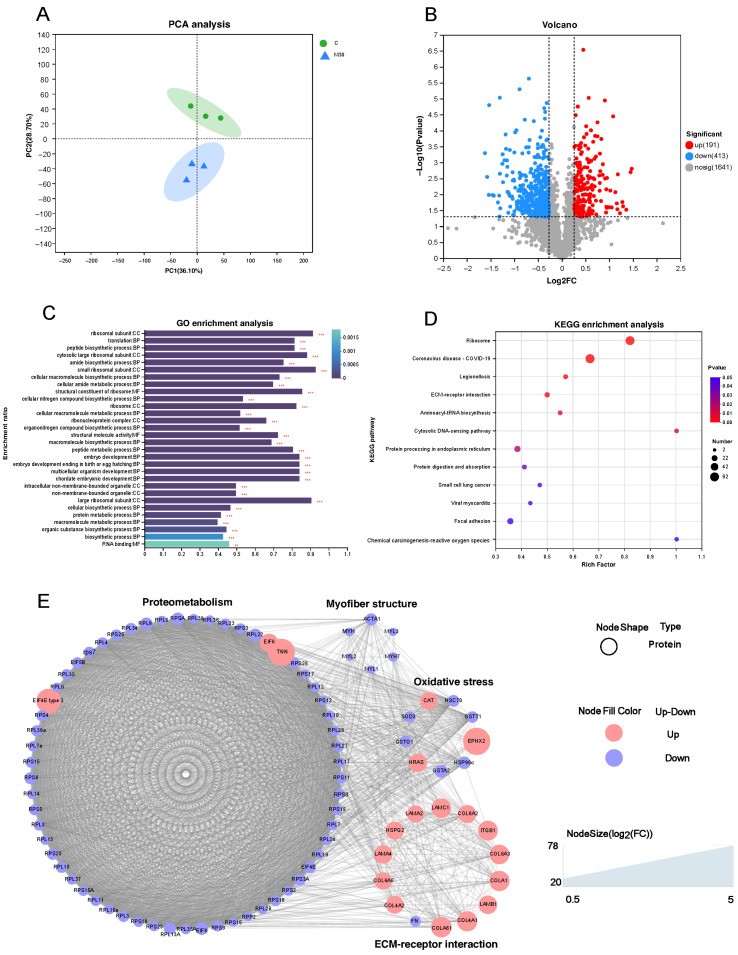
Effects of chronic MC-LR exposure on the proteomic profile of Nile tilapia muscle between M30 group and the control. (**A**) PCA; (**B**) volcano diagram; (**C**) GO enrichment analysis, GO pathways with asterisks represent significant enrichment: ** adjusted *p* < 0.01, *** adjusted *p* < 0.001; (**D**) KEGG enrichment analysis; (**E**) Protein–protein interaction (PPI) network diagram. Red circles represent proteins with increased expression, while blue circles represent proteins with decreased expression. EIF4E, eukaryotic translation initiation factor 4E; EIF4E type 3, eukaryotic translation initiation factor 4E type 3; EIF5B, eukaryotic translation initiation factor 5B; RPL22, 60S ribosomal protein L22; RPS20, 40S ribosomal protein S20; RPS3a, 40S ribosomal protein S3a; RPL13a, 60S ribosomal protein L13a; RPS28, 40S ribosomal protein S28; RPL3L, 60S ribosomal protein L3-like isoform X1; RPL30, 60S ribosomal protein L30; RPL11, 60S ribosomal protein L11; RPL36a, 60S ribosomal protein L36a; RPS6, 40S ribosomal protein S6; RPS18, 40S ribosomal protein S18; RPL28, 60S ribosomal protein L28 isoform X1; RPS8, 40S ribosomal protein S8; RPL10a, 60S ribosomal protein L10a isoform X1; EIF, exportin-1 isoform X1; EIF6, eukaryotic translation initiation factor 6; RPS17, 40S ribosomal protein S17 isoform X1; RPL27, 60S ribosomal protein L27; RPL7, 60S ribosomal protein L7; RPL4, 60S ribosomal protein L4; RPS12, 40S ribosomal protein S12; RPL27a, 60S ribosomal protein L27a; RPL18, 60S ribosomal protein L18; RPL8, 60S ribosomal protein L8; RPL35a, 60S ribosomal protein L35a; RPL23, 60S ribosomal protein L23; RPS9, 40S ribosomal protein S9; RPP0, 60S acidic ribosomal protein P0; RPL34, 60S ribosomal protein L34; RPSA, 40S ribosomal protein SA; RPS26, 40S ribosomal protein S26; RPP2, 60S acidic ribosomal protein P2; RPS7, 40S ribosomal protein S7; RPL12, 60S ribosomal protein L12 isoform X1; RPL9, 60S ribosomal protein L9; RPS19, 40S ribosomal protein S19; RPL26, 60S ribosomal protein L26; RPL7a, 60S ribosomal protein L7a; RPL24, 60S ribosomal protein L24; RPS25, 40S ribosomal protein S25; RPS3, 40S ribosomal protein S3; RPS4, 40S ribosomal protein S4, X isoform; RPS15a, 40S ribosomal protein S15a; RPS2, 40S ribosomal protein S2; RPL5, 60S ribosomal protein L5; RPS15, 40S ribosomal protein S15; RPL36, 60S ribosomal protein L36; RPS5, 40S ribosomal protein S5; RPS10, 40S ribosomal protein S10; RPL10, 60S ribosomal protein L10; RPL14, 60S ribosomal protein L14; RPL13, 60S ribosomal protein L13; RPS23, 40S ribosomal protein S23; RPL6, 60S ribosomal protein L6; RPL38, 60S ribosomal protein L38; RPL19, 60S ribosomal protein L19; RPS16, 40S ribosomal protein S16; RPS30, ubiquitin-like protein fubi and ribosomal protein S30; RPL37, 60S ribosomal protein L37; RPS11, 40S ribosomal protein S11; RPL3, 60S ribosomal protein L3; RPL17, 60S ribosomal protein L17; TNN, tenascin-N; COL4A1, collagen alpha-1(IV) chain isoform X1; LAMA4, laminin subunit alpha-4; COL4A2, collagen alpha-2(IV) chain; HSPG2, basement membrane-specific heparan sulfate proteoglycan core protein isoform X1; LAMA2, laminin subunit alpha-2 isoform X1; DG, dystroglycan; COL6A6, collagen alpha-6(VI) chain isoform X1; LAMC1, laminin subunit gamma-1 isoform X1; LAMB1, laminin subunit beta-1; ITGB1, integrin beta-1 isoform X1; COL6A2, collagen alpha-2(VI) chain; COL6A1, collagen alpha-1(VI) chain; COLA1, collagen alpha-1(I) chain-like precursor; FN, fibronectin isoform X1; COL6A3, collagen alpha-3(VI) chain-like; HRAS, GTPase HRas; EPHX2, bifunctional epoxide hydrolase 2; GSTA2, glutathione S-transferase A2; GSTO1, glutathione S-transferase omega-1; GSTT1, glutathione S-transferase theta-1; CAT, catalase; SOD3, extracellular superoxide dismutase [Cu-Zn]; HSC70, heat shock cognate 71 kDa protein; HSP90α, heat shock protein HSP 90-alpha; HSP90α1, heat shock protein HSP 90-alpha 1; MYL2, myosin regulatory light chain 2; MYH7, myosin-7; MYL3, myosin light chain 3; MYH, myosin heavy chain, fast skeletal muscle; MYL1, myosin light chain 1, skeletal muscle; ACTA1, alpha skeletal muscle actin 1. Data was presented as means ± SD. C, control, 0 μg/L MC-LR; M1, 1 μg/L MC-LR; M3, 3 μg/L MC-LR; M10, 10 μg/L MC-LR; M30, 30 μg/L MC-LR.

As shown in Figure 5A, principal component analysis (PCA) revealed distinct separation between the proteomic profiles of the M30 and control groups. Furthermore, a total of 604 differentially expressed proteins (DEPs) were identified, 191 of which were upregulated and 413 were downregulated proteins (Figure 5B). GO analysis showed that most DEPs were predominantly enriched in the categories of biological process and cellular component, suggesting that MC-LR exposure influenced relevant biological processes and altered cellular components in Nile tilapia muscle (Figure 5C). Specifically, the key enriched biological processes included peptide metabolic process, protein metabolic process, embryo development, multicellular organism development, and cellular biosynthetic process (Figure 5C), which implies that MC-LR exposure likely perturbed protein metabolism and muscle development in Nile tilapia muscle tissue. The main enriched cell components were closely associated with ribosomes, such as ribosomal subunit, cytosolic large ribosomal subunit, small ribosomal subunit, ribosome, ribonucleoprotein complex, and large ribosomal subunit (Figure 5C), suggesting that MC-LR exposure affected the ribosome components, which directly correlates with the downregulation of ribosome pathway proteins and subsequent inhibition of muscle protein deposition. In line with the GO analysis, KEGG analysis revealed that DEPs were also enriched in pathways related to protein metabolism (ribosome, protein processing in endoplasmic reticulum, and protein digestion and absorption), cellular matrix (ECM–receptor interaction and focal adhesion), and oxidative stress (chemical carcinogenesis-reactive oxygen species) (Figure 5D). Protein metabolism is the main pathway, with ECM and oxidative stress as secondary pathways (Figure 5D). The ribosome pathway, the most significant pathway among the 12 enriched pathways, encompassed key DEPs (e.g., EIF4E, RPL22, RPS3a, RPS6) that were predominantly downregulated; this pathway directly mediates protein synthesis—consistent with the decreased myofiber diameter (Figure 4) and increased shear force (Figure 1P) in the M30 group (Figure 5D). In the oxidative stress pathway (chemical carcinogenesis-reactive oxygen species), enriched DEPs (e.g., SOD3, GSTO1, GSTA2) were downregulated, which aligns with the reduced antioxidant capacity (Figure 3A,B,G) and increased lipid/protein oxidation (Figure 3I,J) in the M30 group, ultimately contributing to decreased WHC (Figure 2A,F) and altered meat color (Figure 1L–N). ECM-receptor interaction pathway: Upregulated DEPs (e.g., COL4A1, COL6A1, COL6A2) and decreased MMP-2 activity (Figure 1S) promoted collagen deposition (Figure 1R), which is positively associated with the increased shear force (Figure 1P) in the M30 group. The PPI diagram composed of 97 DEPs was divided four parts, namely, proteometabolism (66 DEPs), ECM-receptor interaction pathway (14 DEPs), oxidative stress (9 DEPs), and myofiber structure (6 DEPs) (Figure 5E). Notably, downregulated DEPs dominated the ribosome and oxidative stress modules, reinforcing that MC-LR exposure suppresses protein synthesis and antioxidant defense—key mechanisms underlying the observed meat quality deterioration.

### 2.6. MC-LR Deposition in Nile Tilapia Muscle

MC-LR exposure significantly increased the MC-LR deposition in the M3, M10, and M30 groups (*p* < 0.05) (Table 1). Although MC-LR deposition was statistically significant but quantitatively low, especially for the M3 and M10 groups (*p* < 0.05) (Table 1). MC-LR concentrations of fish muscle in the control were below the detected limit (0.3 μg/kg), and thus not detected. The MC-LR concentration in M30 group reached approximately 7.5 ng/g (Table 1). Estimated daily intake (EDI) in the M3, M10, and M30 groups were significantly increased (*p* < 0.05), while not exceeding the WHO’s tolerable daily intake (TDI) value (0.04 μg kg^−1^) (Table 1). Moreover, the hazard quotient (HQ) followed a trend similar to that of MC-LR concentration and EDI, with the highest HQ value in the M30 group remaining below 1 (Table 1), suggesting a low risk of adverse effects.

### 2.7. Nutrient Composition of Nile Tilapia Muscle

To further investigate the chronic effects of MC-LR exposure on nutrient composition, a targeted metabolomics analysis focusing on amino acids, fatty acids, and nucleic acid derivatives was performed (Table 2). Exposure to MC-LR (30 μg/L) significantly increased the levels of threonine and aspartic acid, while markedly decreasing the contents of lysine, phenylalanine, valine, glutamate, proline, serine, and total essential amino acids (EAA) (*p* < 0.05) (Table 2). Meanwhile, the M30 treatment exhibited higher contents of C16:0, C18:0, C18:2n − 6, C20:4n − 6, and saturated fatty acid (SFA) and lower contents of C18:1, C20:5n − 3 (EPA), C22:6n − 3 (DHA), monounsaturated fatty acid (MUFA), polyunsaturated fatty acid (PUFA), n − 3/n − 6 PUFA (*p* < 0.05) (Table 2). In addition, the contents of adenosine monophosphate (AMP) and hypoxanthine (Hx) were increased in the M30 treatment, while the levels of adenosine triphosphate (ATP), and inosine (HxR) showed a contrary decrease (*p* < 0.05) (Table 2). Overall, MC-LR exposure led to a deterioration in the muscle nutrient composition of Nile tilapia, characterized by a marked reduction in EAAs and n − 3 PUFAs, indicating that nutritional quality degradation may occur even when toxicological risk is low. Integration of proteomics and targeted metabolomics results revealed that nutrient alterations were consistent with reduced translational capacity and muscle growth, reinforcing Section 2.5, as reduced EAAs and altered nucleotide metabolism strongly support ribosome-centered suppression.

## 3. Discussion

### 3.1. MC-LR Exposure Decreased Sensory Quality and Nutrient Compositions of Nile Tilapia Muscle

Environmental stress from exogenous compounds has been reported to induce deterioration of sensory quality and loss of muscle nutrients in aquatic animals. Meat sensory quality in fish can be assessed based on a range of sensory traits, such as pH value, shear force, water-holding capability (WHC), and meat color [20]. It is well-known that pH value is a key meat parameter that influences the meat sensory properties [3]. The anaerobic glycolysis during the post-mortem period accelerates the accumulation of lactate, leading to a rapid decline in meat pH. In this study, MC-LR exposure had no significant effect on pH_15min_ but significantly reduced pH_24h_. Consistent with this observation, the levels of lactate and glucose-6-P in Nile tilapia muscle were significantly elevated following MC-LR exposure. These results suggested that MC-LR exposure accelerated post-mortem pH decline in Nile tilapia muscle, suggesting that MC-LR may enhance glycolytic potential during the post-slaughter period [21]. Indeed, a previous study demonstrated that prolonged MC-LR exposure promoted a shift toward anaerobic metabolism in the liver of zebrafish, leading to elevated lactate concentration [22].

Notably, a decrease in pH value typically elevates drip loss and cooking loss, thereby reducing the WHC of the meat [21,23]. In this study, MC-LR exposure significantly increased the drip loss and cooking loss of Nile tilapia meat, indicating the decreased WHC. This finding was corroborated by low-field nuclear magnetic resonance (LF-NMR) analysis, which revealed a significant extension of T23 relaxation times in the M30 group. This extension corresponded to an elevated proportion of mobilized water (P23), suggesting that MC-LR exposure facilitated the movement of free water within the muscle tissue, consequently diminishing WHC. Furthermore, a decline in antioxidant capacity can induce lipid peroxidation in cell membranes, compromising their structural integrity and ultimately promoting water loss [24]. Thus, the observed increase in free water in this study may be attributed to MC-LR-induced oxidative damage to cell membranes, specifically via lipid peroxidation. Indeed, MC-LR has been extensively documented to trigger lipid oxidation in diverse tissues, including blood and liver, across various experimental models [10,19].

Muscle color, a key indicator of meat freshness, is evaluated based on L* (lightness), a* (redness), and b* (yellowness), with these parameters primarily linked to lipid oxidation, water retention and light reflection [25]. Myoglobin content is positively correlated with a* and L* values [2]. Excessive ROS induces the oxidation of myoglobin (red) to metmyoglobin (reddish), resulting in reduced a* value and L* values. Concurrently, myoglobin oxidation triggers lipid oxidation, which in turn facilitates the generation of metmyoglobin, contributing to fish meat browning as reflected by elevated b* value [25]. In the present study, MC-LR exposure significantly reduced the values of a* and L* at 24 h, while increasing b* value at 24 h at the same time point, indicating that MC-LR exposure enhanced the oxidation of myoglobin and lipid. This is supported by decreased myoglobin content and increased TBARS levels, which may partially contribute to the decreased L* value at 24 h in Nile tilapia muscle. Furthermore, the reduction in L* value correlates with reduced water content in muscle samples, as lower water content impairs light reflection—an effect reflected by the decreased L*value. Thus, the decreased WHC may partly account for decreased L*value.

The nutrient composition of muscle is a critical parameter for evaluating flesh quality of fish [4]. The present study demonstrates that exposure to MC-LR induces significant alterations in the amino acid, fatty acid, and nucleotide profiles of Nile tilapia muscle, compromising its nutritional value. Specifically, total amino acids (TAA) and essential amino acids (EAAs) exhibited marked reductions in the M30 group compared to the controls, reflecting diminished nutrient content. In terms of fatty acids, MC-LR exposure resulted in a significant increase in saturated fatty acids (SFAs), alongside a concurrent decline in monounsaturated fatty acids (MUFAs). Notably, polyunsaturated fatty acids (PUFAs) showed differential responses: n − 3 series (EPA, DHA) decreased by 15.1% and 13.2%, respectively, while n − 6 arachidonic acid (ARA) increased by 11.9%. This imbalance in n − 3/n − 6 ratios could diminish the nutritional value of tilapia muscle, given the cardioprotective benefits of n − 3 PUFAs [5]. In nucleotide degradation, MC-LR exposure accelerated ATP catabolism, as evidenced by significant elevations in hypoxanthine (Hx) and AMP, accompanied by a decline in IMP. The accumulation of Hx and its derivatives (e.g., HxR) likely impairs meat palatability by reducing the umami attributes associated with IMP retention [25].

Collectively, these results demonstrate that MC-LR exposure compromises the nutritional quality of tilapia muscle through altering amino acid and fatty acid profiles and promoting nucleotide degradation. The observed decreases in EAAs, n − 3 PUFAs, and IMP, coupled with increases in SFAs and Hx, underscore potential risks for both consumer health and aquaculture sustainability. Future studies should investigate long-term exposure outcomes and mitigation strategies aimed at preserving the nutritional integrity of farmed fish. Overall, these findings suggested that MC-LR exposure decreased sensory quality and muscle nutrient content, thereby lowering the meat quality of the Nile tilapia.

### 3.2. MC-LR Exposure-Induced Oxidative Stress of Nile Tilapia Muscle

The increased oxidative stress induced by ROS is the primary mechanism underlying toxic effects of environmental pollutants in fish. MC-LR induces oxidative stress by impairing the antioxidant status in fish. In this study, biochemical analyses revealed that MC-LR exposure significantly reduced the activities of T-AOC (M30), SOD (M30), and GST (M10 and M30) as well as GSH content, while increasing the CAT activity. Most oxidative stress endpoints were the most prominent in the M30 group. Consistent with the biochemical results, proteomic analysis indicated that MC-LR exposure downregulated the protein expression levels of SOD3, GSTO1, GSTT1, and GSTA2 in the Nile tilapia muscle. SOD catalyzes the disproportionation of O_2_- to produce O_2_ and H_2_O_2_, while CAT is known to catalyze the decomposition of H_2_O_2_ into H_2_O and O_2_ [26]. Both SOD and CAT play crucial roles in scavenging free radicals. In the present study, MC-LR exposure significantly increased the CAT activity in Nile tilapia muscle, indicating that the antioxidant system was partly activated to protect cells from free radicals. Notably, however, in addition to SOD, GST also plays a crucial role in antioxidant system by catalyzing the conjugation of GSH with free radicals. Thus, the reduced activities of SOD and GST, along with the decreased GSH content induced by MC-LR, may be responsible for the elevated oxidative stress in Nile tilapia muscle of the M30 group. This was evidenced by increased levels of TBARS and PC, lipid and protein oxidation products, respectively. These results indicated that although MC-LR exposure increased CAT activity in Nile tilapia muscle to mitigate oxidative stress, the induced oxidative stress was not fully alleviated due to the reduced activities of SOD and GST and decreased GSH content caused by MC-LR. Indeed, numerous studies have documented that MC-LR exposure induces oxidative stress by inhibiting the activities of antioxidant enzymes in the liver of other aquatic organisms, including zebrafis [27], *Brycon amazonicus* [28], and the oriental river prawn (*Macrobrachium nipponense*) [29]. Furthermore, GSH plays a critical role in the detoxification metabolism of MC-LR by conjugating it to form GSH metabolites (MC-LR-GSH and MC-LR-Cys) [30]. The decreased GSH content may exacerbate the toxicity of MC-LR to fish muscle, leading to histological damage characterized by reduced muscle fiber diameter, namely, myofiber atrophy. However, the specific molecular mechanisms underlying this process require further investigation. Additionally, MC-LR exposure reduced the contents of serine and proline. Serine has been reported to exert antioxidant and cytoprotective effects by activating antioxidant enzymes in the Nrf2 pathway [3]. Thus, we hypothesized that MC-LR exposure impaired the antioxidant status of Nile tilapia muscle by disrupting amino acid metabolism.

Heat shock proteins (HSPs) are indispensable cellular chaperone proteins in the cells that protect organisms from tissue damage induced by various environmental stressors [31]. HSPs are reported to mitigate ROS-induced oxidative stress by elevating GSH levels and preventing the improper folding of antioxidant enzymes [32]. In the present study, we observed that the levels of HSC70, HSP90α, and HSP90α1 in Nile tilapia muscle were significantly reduced following MC-LR exposure. However, contradictory findings have been reported in previous studies examining the effects of MC-LR exposure on the expressions of HSPs in other aquatic organisms. Acute MC-LR exposure for 168 h upregulated the transcription of HSP90, HSP70, HSP60, and HSP27 in larval zebrafish [33]. Yuan et al. (2016) reported that the expression levels of hsp90, hsp70 and hsp60 in red swamp crayfish were significantly upregulated following MC-LR exposure for 7 days [34]. The discrepancies between the present study and previous findings may be attributed to differences in exposure duration and aquatic species. Short-term environmental stress induced adaptive adjustments in fish, while long-term stress may disrupt the adaptive mechanism [35]. Thus, MC-LR exposure lowered the levels of HSC70 and HSP90α in Nile tilapia muscle due to the loss of cellular adaptive capacity under chronic stress, which may further contribute to antioxidant system dysfunction and ROS accumulation.

### 3.3. MC-LR Exposure Increased Collagen Deposition of Nile Tilapia Muscle

The extracellular matrix (ECM) plays a crucial role in regulating muscle development, which is primarily composed of proteins—with collagen as the major constituent. In this study, 14 DEPs were enriched in the ECM–receptor interaction pathway, specifically including COL14A1, COL4A2, COL6A1, COL6A2, and FN. Consistent with these proteomic data, biochemical results demonstrated increased collagen content in Nile tilapia muscle following MC-LR exposure. Similarly, long-term exposure to MC-LR induced abnormal collagen deposition and fibrosis in mouse ovaries [36]. Perinatal MC-LR exposure increased collagen deposition in the prostate due to decreased activities of MMP-2 and 9 key members of the MMP family, which maintained ECM homeostasis by degrading collagen [37]. Meanwhile, the biochemical results demonstrated reduced activity of MMP-2 in the M30 group. Thus, these findings suggested that MC-LR exposure inhibited collagen degradation by reducing the activities of these two MMPs, ultimately leading to increased muscle collagen deposition in Nile tilapia. Notably, muscle collagen content has been reported to be positively associated with shear force. Accordingly, the increased shear force in MC-LR-exposed fish may be partly attributed to this elevated collagen content.

### 3.4. MC-LR Exposure Decreased Protein Deposition of Nile Tilapia Muscle

The muscle growth of fish is closely associated with protein deposition, which depends on protein synthesis. In this study, the crude protein content in the muscle of Nile tilapia in the M30 group was significantly reduced following MC-LR exposure [38]. Similarly, a previous study demonstrated that dietary cyanobacteria exposure reduced the crude protein content in goldfish (*Carassius auratus*). Ou-Yang et al. (2023) also reported that long-term exposure to *Microcystis aeruginosa*—a common cyanobacterium in algal blooms—resulted in reduced crude protein content in zebrafish muscle [39]. In this study, the DEPs were significantly enriched in pathways related to proteometabolism, including ribosomes, protein processing in the endoplasmic reticulum, and protein digestion and absorption pathways, indicating that chronic high-dose (M30) exposure may disrupt the protein proteometabolism in the muscle of Nile tilapia. Ribosomes are the key organelle responsible for performing protein synthesis, and the ribosome pathway plays essential roles in regulating muscle cells growth by modulating protein synthesis [40]. In this study, numerous protein expression levels in the ribosome pathway, such as EIF4E, EIF5B, RPL22, RPS3a, RPS6, etc., were downregulated after chronic high-dose (M30) exposure (Table S2). Notably, most of them were key components of ribosomes (e.g., RPL22, RPS3a, RPS6), indicating that MC-LR exposure may inhibit the synthesis and assembly of ribosomes, thereby diminishing protein deposition. Moreover, EIF4E is the cap-binding protein, which binds to mRNA and facilitates the recruitment of ribosomes, promoting translation initiation that is a key rate-limiting step of protein synthesis [41]. RPS6 (40S ribosomal protein S6), the first identified substrate of S6K, which is a key signaling molecule that regulates protein synthesis, is thought to be an effector mediating the upregulation of protein synthesis [42]. Therefore, the decreased protein content in ribosome pathway suggested that chronic high-dose (M30) exposure may inhibit the protein synthesis process in ribosomes, which might be closely associated with a decreased protein deposition and myofiber diameter. Notably, shear force, an important sensory parameter, was reported to be negatively associated with muscle fiber diameter and tenderness of fish meat [23]. Thus, chronic high-dose (M30) exposure elevated the shear force of Nile tilapia meat due to the decreased myofiber diameter, resulting in decreased tenderness.

### 3.5. MC-LR Exposure Lowered Myofiber Growth of Nile Tilapia

The muscle growth of fish depends on myofiber growth and development, which are regulated by a variety of proteins. In this study, we identified a regulatory network for muscle growth and development comprising 12 proteins, including MYL1 (myosin light chain 1), MYL2 (myosin regulatory light chain 2), MYL3 (myosin light chain 3), MYO7 (myosin-7), MYH (myosin heavy chain), and ACTA1 (alpha skeletal muscle actin 1).

Myosin is one of the primary structural proteins of muscle that forms the thick filament and is composed of MHC (myosin heavy chain) and MLC (myosin light chain) [43]. A previous study showed that the downregulation of MHC and MLC led to conformational changes in myosin, which reduced the space between thick and thin filaments and decreased myofiber diameter [44]. Actin is also a key structural protein that, together with myosin, forms the thin filaments constituting the myofiber cytoskeleton [45]. Reduced actin protein levels have been shown to decrease myofiber diameter, an effect attributed to the structural disruption of thin filament [46]. The reduced expression of myosin-related and actin-related proteins in the present study may be associated with the decreased muscle fiber diameters of Nile tilapia exposed to MC-LR. Based on the current findings, the decreased contents of these structural proteins could potentially be linked to the MC-LR-induced impairment of protein synthesis (evidenced by downregulated ribosome-related proteins) and elevated oxidative stress (supported by increased lipid/protein oxidation and altered antioxidant enzyme activities). Collectively, these factors may synergistically contribute to muscle atrophy in Nile tilapia, which is manifested by the decreased myofiber diameter and meat yield. However, the direct causal relationships between these molecular changes and myofiber growth inhibition require further validation through targeted functional experiments.

### 3.6. Muscle MC-LR Deposition After Prolonged MC-LR Exposure Was Low and Safe for Consumers

MC-LR exposure has been widely documented to result in MC-LR deposition in the muscle of aquatic animals [47,48]. However, it is important to acknowledge that the current study only quantified free MC-LR in tilapia muscle, which may underestimate the total toxic burden. As demonstrated by Mohamed et al. (2020), bound or conjugated microcystins (e.g., glutathione-conjugated or protein-bound forms) can accumulate in tilapia from aquaculture systems, and these forms are not detected by the standard LC-MS method used in this study [49]. Despite this limitation, the results of the present study showed that the highest estimated daily intake (EDI) of free MC-LR in the M30 group remained lower than the World Health Organization’s tolerable daily intake (TDI, 0.04 μg kg^−1^ d^−1^) for total microcystins [50]. Additionally, the highest hazard quotient (HQ) of free MC-LR in the M30 group was within the range of 0.1 to 1, indicating low adverse effects [47]. Thus, it was concluded that chronic MC-LR exposure (up to 30 μg/L) led to minimal free MC-LR deposition (0.8–7.98 ng/g) in Nile tilapia muscle. However, as highlighted by Poste et al. (2011), the risk associated with microcystin exposure through fish consumption is highly context-dependent, being strongly influenced by factors such as regional fish consumption rates, target fish species, and local cyanobacterial bloom frequency and intensity [51]. Under the tested exposure conditions (environmentally relevant MC-LR concentrations up to 30 μg/L) and the assumed consumption scenario (adult daily intake of 227 g fish muscle), the calculated EDI and HQ values support that the free MC-LR residues in Nile tilapia filets pose a negligible risk to consumers. This conclusion is specific to the experimental settings and species examined herein, and broader extrapolation to other aquaculture systems or consumption patterns should consider the context-dependent nature of microcystin exposure risk as emphasized by Poste et al. (2011) [51].

Importantly, this consumer safety conclusion does not imply an absence of sublethal biological impacts on the fish themselves. As recently demonstrated by Shahmohamadloo et al. (2023a, 2023c) [52,53], fish can meet human consumption safety thresholds while experiencing significant physiological and molecular impairments induced by cyanotoxins, such as hepatotoxicity, metabolic disruption, and histological damage. Consistent with this perspective, the present study observed multiple sublethal effects in Nile tilapia exposed to environmental concentrations of MC-LR, including deteriorated meat quality (decreased pH and water-holding capacity, increased lipid/protein oxidation), impaired antioxidant capacity, inhibited muscle protein synthesis, and reduced myofiber diameter. These findings align with the notion that cyanotoxin exposure can trigger sublethal biological damage in fish even when muscle toxin residues are within safe limits for humans, highlighting the need to consider both consumer health and fish physiological integrity in environmental toxin risk assessments.

Additionally, it is important to acknowledge that the present human health risk assessment has certain limitations, as it relies solely on the WHO’s TDI for MC-LR without incorporating several key factors that may influence real-world risk. First, chronic long-term dietary exposure (exceeding the 60-day duration of this study) could potentially lead to cumulative effects of MC-LR, which warrants further investigation. Second, sensitive populations such as pregnant women, infants, the elderly, and individuals with compromised immune systems may have lower tolerance to MC-LR, and their specific risk was not evaluated herein [16]. Third, natural aquatic environments and aquaculture ponds often experience the co-occurrence of multiple cyanotoxins, which may exhibit synergistic toxic effects that are not captured by single-toxin risk assessment [8,9]. Fourth, non-dietary exposure routes (e.g., skin contact or inhalation during aquaculture activities or recreational water use) could contribute to total MC-LR exposure, but were not considered in the current dietary-focused assessment [49]. These factors highlight the need for more comprehensive risk evaluations in future studies to better reflect real-world exposure scenarios.

## 4. Conclusions

This research revealed that chronic high-dose (M30) exposure diminished the muscle antioxidant capability of Nile tilapia, resulting in declined meat quality (decreased pH value and WHC, increased lipid/protein oxidation) and sublethal biological impairments, including inhibited muscle protein synthesis, reduced myofiber growth, and altered nutrient composition. Interestingly, despite these significant physiological and molecular impacts, low levels of MC-LR deposition in Nile tilapia muscle after prolonged exposure present negligible risk to consumers. This finding aligns with recent evidence that fish can meet human consumption safety thresholds while suffering from cyanotoxin-induced sublethal damage [52,53], emphasizing the importance of distinguishing consumer safety from fish biological integrity. By expanding the perspective beyond human-centric risk assessment to include ecological implications of cyanotoxin exposure, our results provide valuable references for both muscle quality/safety evaluations of farmed fish and the ecological risk assessment of aquatic environments contaminated by microcystins.

## 5. Materials and Methods

### 5.1. Fish Culture

Nile tilapia was purchased from Chengyi Aquaculture Co., Ltd., located in Guangzhou, Guangdong Province, China. Fish culture was conducted following the protocol described in our previous study [54]. Briefly, a cohort of 300 robust juvenile Nile tilapia, with an initial mean weight of 4.57 g (±0.36 g), was randomly allocated to 15 tanks, each with dimensions of 100 cm × 50 cm × 25 cm, with three replicates per treatment. The experimental design consisted of five groups: a control (C, no MC-LR exposure) and four MC-LR-exposed groups at nominal concentrations of 1 (M1), 3 (M3), 10 (M10), and 30 (M30) μg/L. Throughout the 60-day trial, fish were fed twice daily (9:00 AM and 6:00 PM) to apparent satiation using a commercial diet, as outlined in Supplementary Table S1. The aquatic environment in all tanks was carefully regulated to maintain the following conditions: water temperature fluctuating between 26 and 28 °C, dissolved oxygen levels ranging from 7.0 to 7.8 mg/L, and a pH that hovered between 7.2 and 7.8.

MC-LR-lyophilized powder from Taiwan Algae Science Inc (Taoyuan, Taiwan, China). had a purity exceeding 95% as verified by reverse-phase high-performance liquid chromatography (RP-HPLC) (Shimadzu, Kyoto, Japan). To ensure the stability of MC-LR levels throughout the exposure study, the concentration of MC-LR in the exposure water was monitored twice daily using ELISA kits supplied by Mlbio (Shanghai, China). Additionally, every week, half of the water in the exposure tanks was replaced with fresh water that had been supplemented with the appropriate levels of MC-LR to sustain the desired concentrations. The measurements were taken before this replacement of MC-LR level change in water before replacement [54,55].

### 5.2. Fish Sampling

Experimental procedures were carried out in accordance with the guidelines for the “Care and Use of Laboratory Animals in China” by the Animal Ethical and Welfare Committee of the Chinese Society for Laboratory Animals. This work has received approval for research ethics from Guangxi Academy of Marine Sciences, with the relevant approval documentation available upon request. After a 60-day exposure, nine Nile tilapia from each group were carefully selected, with three individuals per replicate tank. The fish were anesthetized by immersion in MS-222 solution (20 mg/L) within a plastic container for 10 min until complete cessation of opercular movement, followed by euthanasia via cranial concussion (a sharp percussive blow to the head) to ensure humane termination. Muscle tissue samples were harvested for biochemical and gene expression analyses. Additionally, muscle tissues from six fish were collected and segmented into two distinct portions. One portion was allocated for proteomic analysis, while the other, measuring 2 mm^3^, was preserved in 4% paraformaldehyde (BL539A, Biosharp, Wuhan, China) for H&E staining. All samples mentioned were flash-frozen using liquid nitrogen and subsequently stored at −80 °C.

### 5.3. Histological Observation

H&E staining was performed with slight modifications based on a previously described method [56]. Initially, the fixed muscle tissues were dehydrated through a graduated ethanol series, equal parts ethanol (No. 64-17-5, Aladdin, Shanghai, China) and xylene (No. 1330-20-7, Aladdin, Shanghai, China), xylene alone, and finally paraffin (No. 8002-74-2, Aladdin, Shanghai, China) (previously, they underwent a dehydration process using a graduated ethanol series, a blend of ethanol and xylene in equal parts, xylene alone, and finally, paraffin). The tissues were then embedded and sectioned into 4-micron-thick slices. Subsequently, the sections were dehydrated prior to rinsing with deionized water. Post-staining, the sections were examined under a light microscope.

### 5.4. Meat Quality Parameters Determination

Muscle tissues were processed by homogenization with deionized water at a ratio of 1:2 (weight/volume) to facilitate pH determination using a pH meter (PH838, SMART SENSOR, Shenzhen, China). The resulting pH value was recorded as the muscle pH.

To measure centrifugal weight loss, muscle tissue samples weighing 1 g, initially denoted as W1, were centrifuged for 30 min (1000× *g*, 4 °C). After centrifugation, the surface moisture was gently blotted with filter papers, and the samples were reweighed (W2). Centrifugal weight loss was calculated as follows: Centrifugal weight loss (%) = (W1 − W2)/W1 × 100.

Commercial assay kits from Jiancheng Biotech (Nanjing, China) were utilized to quantify the levels of glycogen, lactate, and glucose in Nile tilapia muscle. The ELISA Kit was employed to measure the glucose-6-P content.

Myoglobin concentration was determined according to the method described by Shi et al., (2022) [25]. Briefly, 10 g of muscle tissue was homogenized in 10 mL of sodium phosphate buffer, kept on ice for 30 min, and then centrifuged. The absorbance of the supernatant was measured at 525 nm and 700 nm. The calculation for myoglobin content was derived using the formula: Myoglobin content (mg/g) = (A525 − A700) × 2.303 × dilution factor.

Accurately weighed (1 g) ground muscle tissue was thoroughly mixed with 1.5 mL of ice-cold solution containing 0.24 M sucrose and 0.02 M KCl. Following this, the mixture was subjected to homogenization and filtration to extract the residue. The residue was then gently pressed against absorbent paper to remove any surplus liquid before being weighed. The fragmentation index, calculated as a percentage, was derived from the ratio of the weight of the original tissue to that of the residue.

Meat color was measured as L*, a*, and b* values using a colorimeter (LS 172, Linshang, Shenzhen, China). Subsequently, the mixture was homogenized and filtered to obtain the residue. The residue was then gently blotted with absorbent paper to remove any excess liquid prior to weighing. The fragmentation index, expressed as a percentage, was calculated as the ratio of residue weight to the original tissue weight.

Cooking loss was determined as follows: muscle tissue samples weighing 1 g, denoted as W1, were placed within plastic vials and subjected to a cooking process at 70 °C for a duration of 15 min. Once cooked, the samples were allowed to dry off any surface moisture before being re-weighed, now designated as W2. The equation used was as follows: Cooking loss (%) = (W1 − W2)/W1 × 100. Furthermore, water distribution in the muscle tissue was analyzed by low-field nuclear magnetic resonance (LF-NMR) (NMI20-015V-I, NIUMAG, Suzhou, China)following the method described by Wang et al. (2023) [57].

### 5.5. Oxidative Stress Parameters Measurement

Oxidative stress indicators in Nile tilapia muscle were measured using commercial assay kits (Nanjing, China). The parameters analyzed included total antioxidant capacity (T-AOC), glutathione S-transferase (GST), superoxide dismutase (SOD), glutathione peroxidase (GPx), catalase (CAT), protein carbonyl content (PC), glutathione (GSH), glutathione reductase (GR), thiobarbituric acid reactive substances (TBARS), nicotinamide adenine dinucleotide phosphate (NADPH), and total sulfhydryl groups (TS).

### 5.6. Proteomic Profile

#### 5.6.1. Protein Extraction

TMT (Tandem Mass Tag)-based quantitative proteomic analysis was performed with minor modifications to a previously described protocol [58]. Specifically, proteins were extracted from muscle tissues using urea-based lysis buffer (Sangon Biotech, Shanghai, China), which was supplemented with a cocktail of protease inhibitors. Mechanical disruption was performed using a tissue homogenizer for three 40 s cycles. Following this, the samples were incubated on ice for 30 min, with intermittent vortex mixing for 5–10 s at every 5 min interval to ensure thorough lysis. Following centrifugation at 12,000× *g* and 4 °C for 30 min, the supernatant was collected for subsequent protein concentration determination.

#### 5.6.2. Protein Digestion

For each sample, 100 µg of protein was aliquoted and mixed with TEAB buffer (No. 90115, Thermo Fisher Scientific, Waltham, MA, USA). After adding TCEP (No.77720, Thermo Fisher Scientific, Waltham, MA, USA), the mixture was incubated at 37 °C for 60 min. Iodoacetamide (IAM) (No. A39271, Thermo Fisher Scientific, Waltham, MA, USA) was then introduced to a final concentration of 40 mM, and the reaction was carried out at room temperature for 40 min in the dark. The sample was precipitated with six volumes of precooled acetone at −20 °C for 4 h. The pellet was redissolved in 100 µL of 100 mM TEAB buffer, followed by overnight trypsin digestion at 37 °C using an enzyme-to-protein ratio of 1:50.

#### 5.6.3. TMT Labeling

Acetonitrile was added into TMT labeling reagent (No. A44522, Thermo Fisher,Waltham, MA, USA) at ambient temperature. The resulting solution was subjected to centrifugation using a vortex mixer to ensure proper mixing. For each 100 micrograms of polypeptide, an aliquot of the TMT reagent was added, followed by a 2 h incubation period at room temperature to allow for labeling. Subsequently, hydroxylamine was then added and incubated for 30 min. The labeled samples were then pooled into a single tube, equalizing the volume for each, and concentrated using a vacuum concentrator to remove the solvent.

#### 5.6.4. RPLC Separation and LC-MS/MS Analysis

A high pH reverse-phase separation technique was employed to enhance the proteomic coverage by fractionating the samples. The peptide samples were re-solubilized with UPLC loading buffer (2% acetonitrile) and separated in a C18 column (ACQUITY UPLC BEH C18 Column1.7 µm, 2.1 mm × 150 mm, Waters, Milford, MA, USA).

A comprehensive two-dimensional analysis was conducted by liquid chromatography tandem mass spectrometry (Evosep One combined with Obitrap Exploris 480 mass spectrometer) following the stringent standard operating procedures established by Majorbio Technology Co., Ltd. (Shanghai, China). Specifically, the peptide mixture was loaded onto the C18 column (150 μm × 15 cm, Evosep, Odense, Denmark) for liquid phase separation in solvent A (water with 0.1% formic acid) and a linear gradient of solvent B (100% ACN with 0.1% formic acid) at a flow rate of 300 nL/min. The peptides were eluted using the following gradient 0~2 min, 5~5% B; 2~30 min, 5~38% B; 30~40 min, 38~90% B; 40~44 min, 90~90% B.

#### 5.6.5. Protein Identification and Data Analysis

Raw data were analyzed using ProteomeDiscoverer 3.2 software. The precursor mass tolerance was configured to 20 parts per million (ppm), while the fragment mass tolerance was set to 0.02 Daltons. Stringent criteria were applied for peptide identification, with an FDR threshold of not exceeding 0.01. Protein identification was confirmed by the detection of at least one unique peptide match.

Subsequent bioinformatics analyses were performed on the Majorbio Cloud platform, an open-access online resource (accessible at https://cloud.majorbio.com, accessed on 16 March 2025). To discern differentially expressed proteins (DEPs), cutoffs were applied for fold change (values greater than 1.2 or less than 0.83) and a *p*-value threshold of less than 0.05. These DEPs were subjected to GO and KEGG enrichment analyses, which can be accessed at their respective URLs. For protein–protein interaction (PPI) analysis, the String database (Version 11) was employed, leveraging the Nile tilapia-specific database. The visualization was accomplished using Cytoscape software, Version 3.8.2.

### 5.7. MC-LR Deposition Analysis

In accordance with the Chinese national standard method [59], LC-MS was employed to measure the MC-LR levels in tilapia muscle. In short, MC-LR from the samples was extracted using a methanol solution, followed by purification through a solid-phase extraction column. The determination was carried out using a Thermo Scientific Vanquish Flex UHPLC System coupled with a TSQ Triple Quadrupole Mass Spectrometer (Vanquish Flex UHPLC+TSQ) (Thermo Fisher Scientific, Waltham, MA, USA), and quantification was performed using the external standard method.

The tolerable daily intake (TDI) of MC-LR has been established at 0.04 μg kg^−1^ body weight per day the according to World Health Organization, serving as a guideline for safe daily consumption [50]. The hazard quotient (HQ) and daily estimated intake (EDI) were determined following the methodologies outlined in a preceding study [47]. The EDI for MC-LR was calculated (assuming a meal of fish was eaten every day) using the following formula: EDI = (CMC-LR × DI)/BW, where CMC-LR was the concentration of MC-LR in fish muscle, DI is the daily intake of fish for Ontarians (227 g for one meal), and BW represented the body weight of a typical healthy adult, with an assumed average of 70 kg. The hazard quotient (HQ) was calculated to evaluate adverse effects of MC-LR deposition using the following formula: HQ = EDI/TDI. An HQ value of ≤0.1 is deemed negligible, an HQ between 0.1 and 1 is classified as low risk, an HQ between 1 and 10 suggests a moderate hazard, and an HQ exceeding 10 signifies a high level of risk.

### 5.8. Nutrient Composition Analysis

The contents of amino acids, nucleic acids, and fatty acids compounds in Nile tilapia muscle were quantified by targeted metabolomics, as previously described [3]. Detailed methods of targeted metabolomic analysis were shown in Supplementary Text S1.

### 5.9. Statistical Analysis

A one-way ANOVA, complemented by Duncan’s test, was employed to assess significant disparities among various groups, with statistical significance set at *p* < 0.05, utilizing SPSS version 23 software (SPSS Inc., Chicago, IL, USA). For targeted metabolomic analysis (amino acids, fatty acids, and nucleic acid compounds) focusing on pairwise comparisons between the control group (C) and M30 group, Student’s t-test was performed, followed by Benjamini–Hochberg (BH) correction for multiple comparisons to control the false discovery rate (FDR). Statistical significance for these metabolites was defined as adjusted *p* < 0.05.

## Figures and Tables

**Table 1:** The MC-LR deposition and safety assessment of Nile tilapia muscle.

Index	C	M1	M3	M10	M30
MC-LR content (ng/g)	0 ± 0 ^c^	0.8 ± 0.12 ^c^	2.75 ± 0.73 ^b^	7.23 ± 1.51 ^a^	7.98 ± 0.97 ^a^
EDI (μg kg^−1^ body weight day^−1^)	0 ± 0 ^c^	0 ± 0 ^c^	0.01 ± 0 ^b^	0.02 ± 0 ^a^	0.03 ± 0 ^a^
HQ	0 ± 0 ^c^	0.07 ± 0.01 ^c^	0.22 ± 0.06 ^b^	0.59 ± 0.12 ^a^	0.65 ± 0.08 ^a^

Note: Values of same parameters with different letters were significantly different (*p* < 0.05, *n* = 6). C, control, 0 μg/L MC-LR; M1, 1 μg/L MC-LR; M3, 3 μg/L MC-LR; M10, 10 μg/L MC-LR; M30, 30 μg/L MC-LR. EDI (Estimated daily intake) = (C_MC-LR_ × D_intake_)/bw, where C_MC-LR_ is the concentration of MC-LR, D_intake_ is the daily fish consumption for Ontarians (one meal is 227 g), and bw is the body weight of an average healthy adult (assumed 70 kg); HQ (hazard quotient) = EDI/TDI, TDI (tolerable daily intake) is the World Health Organization’s recommended tolerable daily intake of microcystins (0.04 μg kg^−1^ body weight per day).

**Table 2:** The amino acids, fatty acids, nucleic acid compounds composition of Nile tilapia muscle.

Amino Acids (g/100 g)	C	M30	Fatty Acids (mg/100 g)	C	M30
EAA			SFA		
Arg	1.53 ± 0.29	1.4 ± 0.2	C14:0	20.18 ± 2.42	21.78 ± 2.42
His	0.66 ± 0.05	0.6 ± 0.04	C16:0	145.73 ± 10.58 *	159.42 ± 8.73
Ile	0.94 ± 0.06	0.96 ± 0.09	C18:0	48.18 ± 4.35 **	57.59 ± 4.4
Leu	1.68 ± 0.08	1.55 ± 0.1	C20:0	6.6 ± 0.64	7.85 ± 0.66
Lys	2.04 ± 0.11	1.88 ± 0.07 *	C24:0	11.63 ± 1.22	10.73 ± 0.84
Met	0.8 ± 0.06	0.85 ± 0.07	MUFA		
Phe	1.09 ± 0.07	0.94 ± 0.11 *	C16:1	24.76 ± 2.66	26.08 ± 2.39
Thr	1.06 ± 0.09 *	1.18 ± 0.07	C18:1	175.37 ± 9.73	148.58 ± 8.68 ***
Val	2.21 ± 0.07	1.98 ± 0.12 **	C20:1	11.8 ± 1.45	10.26 ± 1.22
NEAA			C24:1	14.81 ± 0.94	16.18 ± 1.59
Ala	1.53 ± 0.15	1.42 ± 0.09	PUFA		
Asp	2.07 ± 0.14	2.27 ± 0.13 *	C18:2n − 6 (LA)	146.67 ± 11.4	129.27 ± 8.18 *
Glu	1.21 ± 0.11	1.04 ± 0.07 *	C18:3n − 3 (ALA)	11.92 ± 1.46	10.73 ± 1.09
Gly	1.26 ± 0.07	1.16 ± 0.08	C18:3n − 6	7.59 ± 0.74	7.23 ± 0.83
Pro	1.03 ± 0.09	0.88 ± 0.06 *	C20:2	11.46 ± 1.37	10.99 ± 1.5
Ser	1.17 ± 0.07	1.03 ± 0.09 *	C20:3n − 6	15.94 ± 0.88	14.79 ± 0.98
Tyr	0.58 ± 0.05	0.61 ± 0.05	C20:4n − 6 (ARA)	33.78 ± 1.52 **	37.8 ± 1.72
EAA	12.01 ± 0.45	11.33 ± 0.35 *	C20:5n − 3 (EPA)	6.94 ± 0.58	5.9 ± 0.69 *
NEAA	8.83 ± 0.22	8.42 ± 0.22	C22:6n − 3 (DHA)	16.15 ± 1.21	14.03 ± 1.27 *
TAA	20.84 ± 0.33	19.75 ± 0.45 **	C22:5n − 6	35.96 ± 2.76	33.04 ± 1.55
			C22:5n − 3	6.44 ± 0.4	6.19 ± 0.49
Nucleotides	C	M30	∑SFA	232.32 ± 14.19 *	257.37 ± 10.88
ATP (µmol/100 g)	0.37 ± 0.07	0.27 ± 0.07 *	∑MUFA	226.74 ± 10.25	201.1 ± 10.74 **
ADP (µmol/100 g)	2.35 ± 0.16	2.17 ± 0.13	∑PUFA	292.84 ± 10.93	269.97 ± 5.7 **
AMP (µmol/100 g)	4.67 ± 0.32 *	5.3 ± 0.37	∑n − 3PUFA	41.45 ± 2.31	36.85 ± 1.64 **
IMP (µmol/100 g)	45.57 ± 6.25	40.45 ± 4.95	∑n − 6PUFA	239.93 ± 12.76	222.14 ± 5.98 *
HxR (µmol/100 g)	0.63 ± 0.05	0.52 ± 0.07 *	n − 3/n − 6PUFA	0.17 ± 0.02	0.17 ± 0.01
Hx (µmol/100 g)	2.26 ± 0.16 *	3.08 ± 0.44	PUFA/SFA	1.27 ± 0.11	1.05 ± 0.04 **

Notes: EAA: essential amino acids; NEAA: nonessential amino acids; TAA: sum of total amino acids. EAA = Lys + Met + Thr + Val + Leu + Ile + Phe + Try + His + Arg. NEAA = Glu + Asp + Ala + Ser + Tyr + Pro + Gly + Cys. FA, fatty acids; SFA, saturated fatty acids; MUFA, monounsaturated fatty acids; PUFA, polyunsaturated fatty acids. n − 3/n − 6: the ratio of n − 3 polyunsaturated fatty acids to n − 6 polyunsaturated fatty acids. ∑n − 3 PUFA (C18:3n − 3, C20:5n − 3, C22:6n − 3, C22:5n − 3). ∑n − 6 PUFA (C18:2n − 6, C18:3n − 6, C18:2n − 6, C20:4n6, C18:2n − 6, C22:5n − 6). ATP, adenosine triphosphate; ADP, adenosine diphosphate; AMP, adenosine monophosphate; IMP, inosine monophosphate; HxR, hypoxanthine riboside (inosine); Hx, hypoxanthineData of muscle was presented as means ± SD (n = 6). Statistical significance was determined by Student’s *t*-test followed by Benjamini–Hochberg (BH) correction for multiple comparisons. Values in the same row with asterisks are significantly different: * adjusted *p* < 0.05, ** adjusted *p* < 0.01, *** adjusted *p* < 0.001.

## Data Availability

The original contributions presented in this study are included in the article. Further inquiries can be directed to the corresponding author(s).

## Supplementary Materials

The following supporting information can be downloaded at https://www.mdpi.com/article/10.3390/toxins18010039/s1. Table S1. The composition of experimental diet ingredients (g/kg); Table S2. The list of key differentially expressed proteins (DEPs); Text S1. Detailed methods of targeted metabolomic analysis.

## Abbreviations

The following abbreviations are used in this manuscript:

C	a control group
M1	MC-LR at concentrations of 1 μg/L
M3	MC-LR at concentrations of 3 μg/L
M10	MC-LR at concentrations of 10 μg/L
M30	MC-LR at concentrations of 30 μg/L
PUFA	polyunsaturated fatty acids
WHC	water-holding capacity
MCs	microcystins
T-AOC	total antioxidant capacity
GST	glutathione S-transferase
SOD	superoxide dismutase
GPx	glutathione peroxidase
CAT	catalase
PC	protein carbonyl content
GSH	glutathione
GR	glutathione reductase
TBARS	thiobarbituric acid reactive substances
NADPH	nicotinamide adenine dinucleotide phosphate
TS	total sulfhydryl groups
DEPs	differentially expressed proteins
PPI	protein–protein interaction
TDI	tolerable daily intake
HQ	hazard quotient
EDI	estimated intake
LDH	lactate dehydrogenase
HSPs	heat shock proteins
ECM	extracellular matrix
MYL1	myosin light chain 1
MYL2	myosin regulatory light chain 2
MYL3	myosin light chain 3
MYO7	myosin-7
MYH	myosin heavy chain
ACTA1	alpha skeletal muscle actin 1
MHC	myosin heavy chain
MLC	myosin light chain
