# Supplementary material for: TMT Quantitative Proteomics Reveals the Molecular Mechanism Behind Meat Quality Changes in Nile Tilapia Exposed to Environmental Concentrations of Microcystin-LR
Source: Toxins (Basel). 2026 Jan 12;18(1):39. doi: 10.3390/toxins18010039 (PMC12846687; doi:10.3390/toxins18010039)
Supplement: Supplementary file 1 [file toxins-18-00039-s001.zip › toxins-4045963-supplementary.pdf]

TMT quantitative proteomics reveals the molecular mechanism behind meat quality changes of Nile tilapia exposed to environmental concentrations of microcystin-LR

Yichao Li [1,2,†], Huarong Xiao [3,†], Jun Xie [3], Liping Liu [2], Fajun Jiang [1], Jingqiu Liao [1,*] and Ermeng Yu [1,*]

[1] Guangxi Key Laboratory of Marine Environmental Science, Guangxi Academy of Marine Sciences, Guangxi Academy of Sciences, Nanning 530007, China
[2] China-ASEAN Belt and Road Joint Laboratory on Mariculture Technology (Shanghai), Shanghai Ocean University, Shanghai 201306, China
[3] Pearl River Fisheries Research Institute, Chinese Academy of Fishery Sciences, Guangzhou 510380, China
* Correspondence: ljq1814@gxas.cn (J.L.); yem@prfri.ac.cn (E.Y.)
† These authors contributed equally to this work.

Supplementary materials

Table S1. The composition of experimental diet ingredients (g/kg).

Ingredients	g/kg	Nutrients content	(%)
soybean meal	300	crude protein	30.79
cottonseed meal	120	crude fat	5.97
rapeseed meal	200	moisture	10.03
wheat flour	120		
rice bran	108		
wheat bran	80		
Soybean oil	30		
choline chloride	2		
mineral mixture	20		
vitamin mixture	20		

Note: Mineral mixture: iron 5 g/kg, copper 100 mg/kg, zinc 1.5 g/kg, manganese 0.5 g/kg, magnesium 20 g/kg, iodine 15 mg/kg, cobalt 5 mg/kg, selenium 5 mg/kg, and sodium, calcium, etc. Vitamin mixture: vitamin A 180000 IU/kg, vitamin D3 40,000 IU/ kg, vitamin E 1.6 g/kg、vitamin K3 0.0.5 g/kg, vitamin B1 0.2 g/kg, vitamin B2 0.25 g/kg, vitamin B6 0.2 g/kg, vitamin B12 0.65 mg/kg, vitamin C 2.5 g/kg, niacin 0.65 g/kg, calcium pantothenate 0.65 g/kg, folic acid 0.03 g/kg, Inositol 1 g/kg, Biotin H 8.3 mg/kg.

Table S2. The list of key differentially expressed proteins (DEPs).

Text S1. Detailed methods of targeted metabolomic analysis.

The amino acids analysis was performed by LC-MS (n=6). Muscle samples (20mg) were treated with 500 μL extract (50% acetonitrile water). After ground for 6 min in a grinding machine (-10 ℃, 50 Hz) and centrifugation for 5 min (4℃, 13000 rcf), 40 μL of supernatant was diluted with 160 μL of 50% acetonitrile water and centrifugated for 5 min (4℃, 13000 rcf) to obtain supernatant for analysis. The analytical conditions were as follows, UPLC: ExionLC AD system; column, Waters BEH Amide (100×2.1 mm,1.7 μm); column temperature, 35 ℃; injection volume, 2μL; flow rate, 1 mL/min; The mobile phase was composed of (A) 0.4% formic acid 20mM ammonium formate-95% acetonitrile solution and (B) 0.4% formic acid with 20mM ammonium formate-5% acetonitrile solution. The following gradient procedures were used: 1~2.6 min, 90% A and 10% B; 2.6~3.5 min, 85% A and 15% B；3.5~4 min, 70% A and 30% B；4~4.1 min, 70% A and 30% B; 4.1~6 min, 100% A. Mass spectrometry conditions (MS) were as follows, a heated ESI source, AB SCIEX QTRAP 6500+; Curtain Gas (CUR), 35; Collision Gas (CAD), Medium; IonSpray

Voltage(IS), 5500; Temperature (TEM), 350; Ion Source Gas1 (GS1), 70; Ion Source Gas2 (GS2), 70. Individual ion fragments were automatically identified and integrated in the AB Sciex quantification software OS , version 3.0. The linear regression standard curve was drawn using the mass spectral peak area of the analyte and the concentration of the analyte as the abscissa. The contents of amino acids were calculated by substituted MS peak area of samples into the linear equation.

The fatty acids analysis was performed by GC-MS (n=6). Muscle samples (20mg) were treated with 1 mL solution (dichloromethane: methanol (v/v=1:1)) and a steel ball, and then ground for 3 min. After ultrasonic processing for 15 min at 4 °C, stand for 15 min at -20 °C, and centrifugation at for 10 min (4 ℃, 13000 rcf), 500 μL supernatant was dried under nitrogen. Then, 0.5 mL methanol solution (0.5 mol/L) was added into the tube, followed by vortex for 30s, and water bath for 0.5 h at 60 ℃. Subsequently, 0.5 mL of hexane was added into the mixture, followed by vortex for 30s at 4 ℃ and centrifugation at 13,000 rcf for 10 min to obtain 100 uL supernatant for GC-MS analysis. The analysis was carried out using gas chromatography (Agilent 8890-7000D, CA, USA) equipped with a fused silica capillary column (20m, 0.18mm, 0.2μm, Agilent, Santa Clara, CA, USA). High purity helium gas (99.99%) was used as the carrier gas. Flow rate, 1.0 mL/min; injection volume, 2μL; The following heating gradient procedures were used: the initial temperature of the column temperature box is 80℃, maintained for 0.5 min; rise to 175 ℃ at 70 ℃/min; rise to 230 ℃ at 8 ℃/min, maintained for 1 min; Run running 80℃, maintained for 2 min. Mass spectrometry conditions (MS) were as follows: electron bombardment of an ion source (EI); ion source temperature, 230 ℃; four-level bar temperature, 150 ℃; transmission line temperature, 240 ℃; electron Energy, 70 eV; Ion scanning mode (SIM). Each ion fragment was automatically identified and integrated with masshunter quantitative software (Agilent, version No.: v10.0.707.0) with default parameters and assisted in manual inspection. The linear regression standard curve was drawn using the mass peak area as the longitudinal coordinate and the concentration of the analyte as the abscissa. Fatty acids concentration of muscle samples was calculated by substituting the mass spectrum peak area of the sample analyte into the linear equation.

Nucleic acid-related compounds were measured by high-performance liquid chromatography (HPLC) (n=6). All the operations were conducted below 4°C. The samples of Nile tilapia muscle were weighed 5.00 g and homogenized with 10 ml of cold perchloric acid, which volume fraction was 10%, by means of a FM-200 homogenizer (Fluko Equipment Shanghai Co. Ltd, Shanghai, China) for 30 s, and centrifuged at 10,000 g for 15 min below 4°C. The precipitation was centrifuged under the same conditions after washing by 5 ml cold perchloric acid with a volume fraction of 5%, and then repeated this step twice and combined supernatant. The combined supernatant was adjusted pH to 6.5 with potassium hydroxide solution at 1 and 10 M concentrations, and then stilled for 30 min. The supernatant was diluted with high purity water to 10 ml and filtered with 0.45-μm membrane. Then, the supernatant was detected and analyzed using standards (Sigma Chemical Co., St. Louis, MO, USA) and high-performance liquid chromatography (Agilent 1100 HPLC, Santa Clara, CA, USA) equipped with a liquid chromatography column (C18, 4.6mm×250mm, CA, USA). Qualitative and quantitative analysis were done on the basis of the retention time of standard compounds and peak area of samples, respectively. The contents of nucleic acid compounds were expressed as μmol/100 g dry matter (DM).
